# Volatile organic compounds and cancer risk assessment in an intensive care unit

**DOI:** 10.1007/s00484-024-02701-w

**Published:** 2024-07-18

**Authors:** Sanaz Lakestani

**Affiliations:** Scientific Industrial and Technological Application and Research Center, Bolu Abant Izzet Baysal University, 14030 Golkoy, Bolu, Turkey

**Keywords:** Intensive care unit, Monitoring, Volatile Organic compounds, Cancer Risk, Hazard Index

## Abstract

Changes caused by air-cleaning devices in the amounts of volatile organic compounds in an intensive care unit were monitored in the study. The cancer risk and hazard index were calculated. The measurements were made for one month at isolated room and two different points and times in the intensive care unit. According to the sampling program, the air-cleaning devices were turned off in weeks 1 and 4 and turned on in weeks 2 and 3. Volatile organic compounds were collected by active sampling. Samples were analyzed by a thermal desorber coupled to a gas chromatography-mass spectrometry instrument with selective ion monitoring. The results showed that the concentrations of benzene, toluene, and o-xylene decreased by about 70% after the air-cleaning devices were installed. The cancer risk assessment for naphthalene was recorded at the highest level of cancer risk (Class A). The hazard index value of naphthalene was recorded at the harmful level when air-cleaning devices were not installed. The concentrations of benzene (*p* = 0.01), toluene (*p* = 0.02), ethylbenzene (*p* = 0.02), styrene (*p* = 0.01), and m, p-xylene (*p* = 0.04) before the air-cleaning devices were installed were significantly different from those recorded when the air-cleaning devices were turned on.

## Introduction

Indoor air pollutants have many sources, including outdoor air, human bodies, wallpaper, carpet, and air conditioning systems (Lindemann et al. 1982; Pastuszka et al. 2000; Hargreaves et al. 2003; Kalogerakis et al. 2005; Tseng et al. 2011; Hospodsky et al. 2012; Yang et al. 2014; Huang et al. 2017; Xu et al. 2017). The most important sources of indoor air pollution are volatile organic compounds (VOCs). Benzene, toluene, ethylbenzene, meta-, para-, and ortho-xylene are emitted from various natural and anthropogenic sources (Li et al. 2020). Due to the health risks posed by exposure to VOCs, it is important to determine the levels of these compounds as well as of other pollutant types when determining indoor air quality (Adgate et al. 2004; Dodson et al. 2007). Indoor air quality is especially important in public buildings.

In hospitals, indoor air quality is affected by the activities carried out, and healthcare workers and patients may be exposed to a wide range of chemical compounds emitted from different products like disinfectants and sterilants (ethylene oxide, glutaraldehyde, formaldehyde, alcohols, etc.), anesthetic gases, laboratory or pharmaceutical products (Huang et al. 2017), and outdoor air (e.g., vehicle emissions) (Nicholas 2014).

The air quality requirements in healthcare facilities vary depending on the health function and can even differ from room to room based on its use. Areas like operating rooms, intensive care units, and isolation rooms require high-efficiency filtration to protect patients, staff, and visitors. Other areas require the removal of gaseous contaminants, chemicals, and odors to create a safer and more pleasant workplace (Settimo and Gola 2017).

Extensive studies have been conducted on indoor air quality (IAQ) and volatile organic compounds (VOCs) in office environments, but limited information is available on the composition and concentration of VOCs in hospital environments (Salonen et al. 2009; Rautiainen et al. 2019). The building materials generally used in hospital and office environments are similar. There are many other sources of VOCs in hospitals that are often interconnected. For example, in healthcare activities, large quantities of alcohol-based hand sanitizer are used in all sectors, as well as skin antiseptics (Rautiainen et al. 2019). Operating rooms, intensive care units, and pathology are areas where additional VOCs are found. Other areas where VOCs are present include wards and emergency rooms. Disinfectants like ethanol and 2-methyl-2-propanol, as well as medical and laboratory chemicals like x ylenes, are commonly used in the pathology unit for tasks such as deparaffinizing tissues before staining or extracting DNA (Rautiainen et al. 2019). The composition and concentration of VOCs vary between hospitals, office buildings, and schools. Many emission sources can result in high concentrations of VOCs indoors. Description of the composition and frequency of VOCs in the hospital indoor air is especially important for healthcare workers. Constant exposure to this complex mixture of VOCs through inhalation and skin contact can have significant health implications. 2023 Riveron Healthcare workers were exposed to higher TVOC compared to other hospital workers (LeBouf et al. 2014; Riveron et al. 2023). Nursing assistants and practical nurses were exposed to personal TVOC concentrations of 9200 µg/m3 and 8700 µg/m3, respectively, compared to clinical laboratory technicians who were exposed to 2000 µg/m3 personal TVOC concentrations (Health and Safety Executive 2020; Riveron et al. 2023). Exposure to VOCs can cause many adverse health effects (Riveron et al. 2023), including deficits in lung function, chronic respiratory disease, lung cancer, heart disease, developmental disorders, and damage to the brain, nervous system, liver, or kidneys (Wang and Pinkerton 2007; Weschler 2011; Lakestani et al. 2013). The World Health Organization (WHO) reported that the world’s top three causes of death are cardiovascular, respiratory, and neonatal conditions (Maung et al. 2022). Studies have shown that short- or long-term exposure to toxic pollution is associated with an increased risk of cardiovascular and respiratory diseases, premature death, and cancer (Li et al. 2020).

The carcinogenic classification published by the International Agency for Research on Cancer (IARC) in 2012 is shown in Table 1. According to this classification, VOCs are classified into four groups: carcinogenic for humans (1), a probable carcinogen (2 A), a possible carcinogen (2B), and non-carcinogenic (3) (International Agency for Research on Cancer 2012).

**Table 1:** Unit risk amount of VOCs and carcinogenic classification published by the IARC

VOC	IARC
Benzene	1
Styrene	2 A
Ethylbenzene	2B
Naphthalene	2B
Xylenes	3

There are many studies examining the impact of indoor air quality on healthcare workers (Glumbakaite et al. 2003; Hellgren and Reijula 2011; Rautiainen et al. 2019; Riveron et al. 2023). According to the studies, 50% of healthcare workers in operating rooms experienced upper respiratory tract symptoms, 40% complained of skin reactions, and 20% complained of headaches (Rautiainen et al. 2019; Riveron et al. 2023). Airborne benzene, commonly found in many everyday products used indoors by humans, is a toxic compound that can rapidly spread into indoor air. Control of benzene is essential, as it is frequently present in ambient air due to insufficient ventilation, posing a threat to human health at high concentrations (Isinkaralar 2023).

To reduce VOC concentration in ICUs, several methods can be employed. One approach is to use air recirculation devices that contain activated carbon filters (Carroll and Kirschman 2022; Isinkaralar et al. 2023). The use of Air-cleaning devices (ACDs) in the indoor environment can help control a range of pollutants such as allergens, particulate matter, bioaerosols, and odors (Babaei et al. 2017). Regular monitoring, ventilation, and regularly maintaining air cleaning devices and replacing filters as needed to ensure optimal performance in reducing VOC concentrations (Rawat and Kumar 2023). Another way to decrease the risk of indoor air pollution is by controlling the sources of the pollutants.

There is little research on chemical pollution and its effects in hospitals in Turkey, especially among healthcare workers. In the present study, to decrease indoor air pollution an Aerte AD 2.0 (Aerte Ltd., UK) ACD was used. This device works on the principle of generating hydroxyl radicals (Aerte 2011). Hydroxyl radicals (OH•) are known as “nature’s disinfectant,” and the activity of hydroxyl radicals is essential to life. These radicals are not usually present in enclosed spaces (Nicholas 2014).

VOCs in the ICU can be measured using various techniques, including ion mobility spectrometry (IMS), GC-MS, and electronic noses (Steinbach et al. 2019; Van Oort et al. 2021). In this study Samples collected were analyzed by a thermal desorber coupled to a gas chromatography-mass spectrometry (GC-MS) instrument. Qualitative analysis of samples was carried out to identify any additional compounds. The aim of the study was to evaluate the effectiveness of ACDs in reducing VOCs and cancer risk in an intensive care unit (ICU).

## Materials and methods

### Sampling area

The study was conducted in the ICU of a hospital in Bolu, Turkey. There were 12 beds at two different points, 11 beds at one point, and 1 bed in an isolated room. The ICU contained patient monitoring, respiratory and cardiac support, pain management equipment; emergency resuscitation devices; and other life support equipment. The floor space of the ICU was about 250 m^2^ and high-efficiency particulate air (HEPA) filters were located above the 12 beds in the two rooms.

### VOC sampling

Sampling was performed at two different points in the ICU and isolated room on three days (Monday, Wednesday, and Friday) twice a day, in the morning and afternoon, for a month, in weeks 1 and 4 when the ACD was turned off and in weeks 2 and 3 when the ACD was turned on. On every sampling day, all changes in the ICU, like the number of persons, activities, disinfecting, and cleaning were recorded.

### Analyses of VOCs

**Fig. 1:**
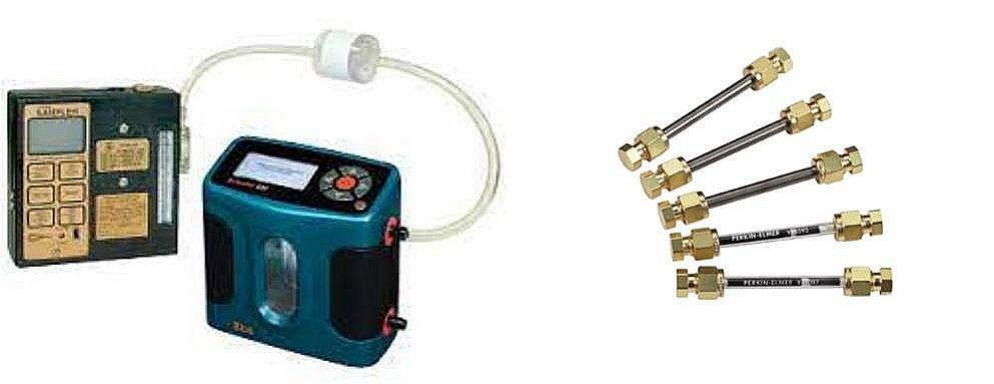
Air sampling pump (SKC Pump), SKC defender 510 calibrator, and sampling tubes

Air samplers were installed on a flat and horizontal surface about 1.5 m above the floor. Samples were collected in Tenax TA sorbent-filled tubes using a personal air sampling pump (SKC Pump) at a flow rate of 80–85 mL min^− 1^ over 45 min (Fig. 1) (Lakestani et al. 2013). The pumping flow rate was set before each sampling session, using an SKC Defender 510 calibrator, and checked at the end of the session to ensure that the flow rate had not changed (Fig. 1). The sample tubes were cleaned and conditioned. Indoor temperature and relative humidity were measured. The sampling of VOCs in the study was based on the US EPA method TO-17 (EPA 1999). After VOC sampling, the sorbent tubes were immediately capped, put in a cooler box, then transferred to the laboratory, and stored at -20 °C until analysis. All the analyses were carried out at the Scientific Industrial and Technological Applications and Research Center (SITARC) of Bolu Abant Izzet Baysal University.

**Fig. 2:**
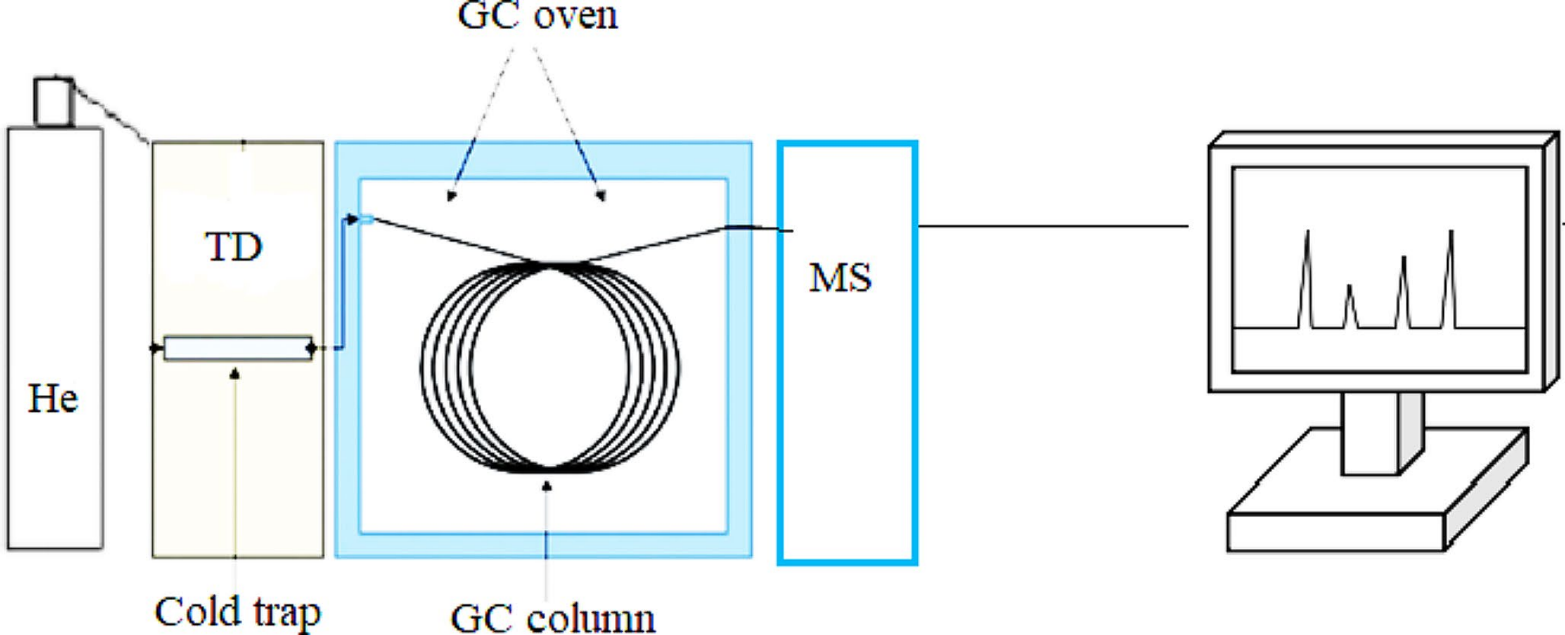
Thermal desorber, gas chromatography-mass spectroscopy schematics

The samples collected were analyzed by thermal desorber (Markes-TD-100) and GC (Thermo Scientific 1300) MS (Thermo Scientific ISQ- QC) instruments by selective ion monitoring (SIM). Helium was used as the carrier gas, and the column used was a fused-silica capillary column (TG-624, ID: 0.25 mm, Length: 30 m, Film: 1.4 μm). GC temperature programming was maintained from 65 °C to 170 °C with a constant rise of 5 °C.min^− 1^, and further, the temperature was increased by 10 °C.min^− 1^ up to 220 °C and again held isothermally at 220 °C for 5 min. MS was performed at 70 eV, the interface temperature was 230 °C, and the ion source temperature was 150 °C (Fig. 2). The mass spectrum of the GC-MS was interpreted using the database of the National Institute of Standards and Technology (NIST), containing more than 62,000 patterns.

## Results and discussion

Since this study was conducted in the intensive care unit, entering was a bit difficult because it is the most important, sensitive, and critical place in the hospital. Sampling was not possible after 5 pm, as the evening hours were designated as resting time for the patients and only doctors and nurses were allowed to enter.

### Identification of VOCs

To verify the accuracy of the collected data, the same sampling process was conducted simultaneously in an isolated room within the intensive care unit. This room was vacant, with no external factors present (Table 2).

**Table 2:** The mean of VOCs in the isolated room when the air-cleaning device (ACD) was on or off

	^a^ACD off	ACD on	^b^ACD off
*N	9	18	9
	**µg/m** ^**3**^		
**Benzene**	0.89	0.02	0.56
**Toluene**	8.47	0.08	5.20
**Ethylbenzene**	2.26	0.05	1.29
**m, p-Xylene**	3.69	0.01	0.22
**o-Xylene**	2.12	0.56	0.94
**Styrene**	3.72	0.30	1.62
**Isopropylbenzene**	1.80	0.87	0.25
**n-Propylbenzene**	3.19	0.73	0.95
**1,2,4-Trimethylbenzene**	1.23	0.88	0.57
**1,3,5-Trimethylbenzene**	4.55	1.73	1.33
**sec-Butylbenzene**	8.65	2.02	3.81
**4-Isopropyltoluene**	3.73	0.93	1.71
**n-Butylbenzene**	1.20	0.26	0.61
**Naphthalene**	2.70	0.25	1.97
**TVOC**	48.21	8.69	21.03

^a^Before the devices were installed, ^b^When the devices were turned off after a two-week “on” period,

*N: Number of samples

Table 3 summarizes the mean, standard deviation (SD), maximum, and minimum concentrations of 36 samples of VOCs (µg/m^3^) collected from the ICU in June-July 2018. The mean values of benzene (2.69 µg/m^3^), toluene (25.88 µg/m^3^), ethylbenzene (6.38 µg/m^3^), m, p-xylene (4.94 µg/m^3^), o-xylene (5.15 µg/m^3^), and styrene (10.07 µg/m^3^) in the ICU before the air-cleaning devices were installed were highest. Toluene was the most abundant compound, followed by styrene.

**Table 3:** The mean, SD, max., and min. concentrations of VOCs when the air-cleaning device (ACD) was on or off

	*N	Mean ± SD	Min.	Max.		*N	Mean ± SD	Min.	Max.
		µg/m^3^					µg/m^3^		
	**Benzene**					**Toluene**			
^**a**^ **ACD off**	9	2.58 ± 1.57	0.76	5.37	^a^ACD off	9	25.30 ± 11.08	8.27	35.60
**ACD on**	18	0.80 ± 0.65	0.01	2.00	ACD on	18	6.22 ± 4.61	0.02	13.39
^**b**^ **ACD off**	9	0.99 ± 0.79	0.02	1.87	^b^ACD off	9	17.27 ± 15.73	0.01	36.81
	**Ethylbenzene**					**m,p-Xylene**			
^**a**^ **ACD off**	9	6.15 ± 2.33	1.71	8.62	^a^ACD off	9	5.66 ± 3.52	2.36	11.60
**ACD on**	18	1.75 ± 1.17	0.01	3.54	ACD on	18	2.72 ± 1.93	0.004	5.85
^**b**^ **ACD off**	9	1.76 ± 1.25	0.09	2.94	^b^ACD off	9	0.52 ± 0.44	0.01	1.08
	**o-Xylene**					**Styrene**			
^**a**^ **ACD off**	9	5.10 ± 4.10	1.06	13.59	^a^ACD off	9	22.52 ± 19.62	0.38	49.95
**ACD on**	18	1.81 ± 1.64	0.07	4.56	ACD on	18	3.49 ± 2.24	0.30	6.85
^**b**^ **ACD off**	9	1.11 ± 0.92	0.02	2.30	^b^ACD off	9	4.83 ± 7.08	0.87	18.91
	**Isopropylbenzene**					**n-Propylbenzene**			
^**a**^ **ACD off**	9	2.91 ± 1.71	0.55	5.13	^a^ACD off	9	16.59 ± 17.00	4.19	45.79
**ACD on**	18	1.20 ± 0.92	0.22	2.88	ACD on	18	2.80 ± 1.35	1.23	4.96
^**b**^ **ACD off**	9	1.06 ± 0.80	0.02	2.15	^b^ACD off	9	1.02 ± 1.45	0.05	3.00
	**1,2,4-Trimethylbenzene**					**1,3,5-Trimethylbenzene**			
^**a**^ **ACD off**	9	6.02 ± 4.76	0.99	12.97	^a^ACD off	9	8.71 ± 3.52	4.94	14.73
**ACD on**	18	1.37 ± 1.06	0.37	3.13	ACD on	18	3.05 ± 2.61	0.44	9.47
^**b**^ **ACD off**	9	0.76 ± 0.25	0.51	1.21	^b^ACD off	9	0.62 ± 0.42	0.12	1.17
	**sec-Butylbenzene**					**4-Isopropyltoluene**			
^**a**^ **ACD off**	9	26.41 ± 18.48	3.91	48.18	^a^ACD off	9	7.40 ± 4.07	3.90	12.88
**ACD on**	18	4.39 ± 2.08	1.82	7.56	ACD on	18	2.11 ± 1.54	0.76	5.73
^**b**^ **ACD off**	9	0.50 ± 0.13	0.29	0.67	^b^ACD off	9	3.80 ± 1.83	2.06	6.04
	**n-Butylbenzene**					**Naphthalene**			
^**a**^ **ACD off**	9	2.13 ± 1.73	0.61	5.06	^a^ACD off	9	4.92 ± 3.36	1.27	8.76
**ACD on**	18	1.39 ± 1.44	0.15	3.96	ACD on	18	0.54 ± 0.12	0.44	0.75
^**b**^ **ACD off**	9	1.66 ± 1.07	0.33	3.13	^b^ACD off	9	4.28 ± 2.57	0.44	7.81
	**TVOC**								
^**a**^ **ACD off**	9	136.78 ± 61.82	53.34	215.74					
**ACD on**	18	38.40 ± 16.20	9.50	68.20					
^**b**^ **ACD off**	9	36.44 ± 15.81	14.66	57.85					

^a^Before the devices were installed, ^b^When the devices were turned off after a two-week “on” period, *N: Number of samples

### Statistical analysis

**Fig. 3:**
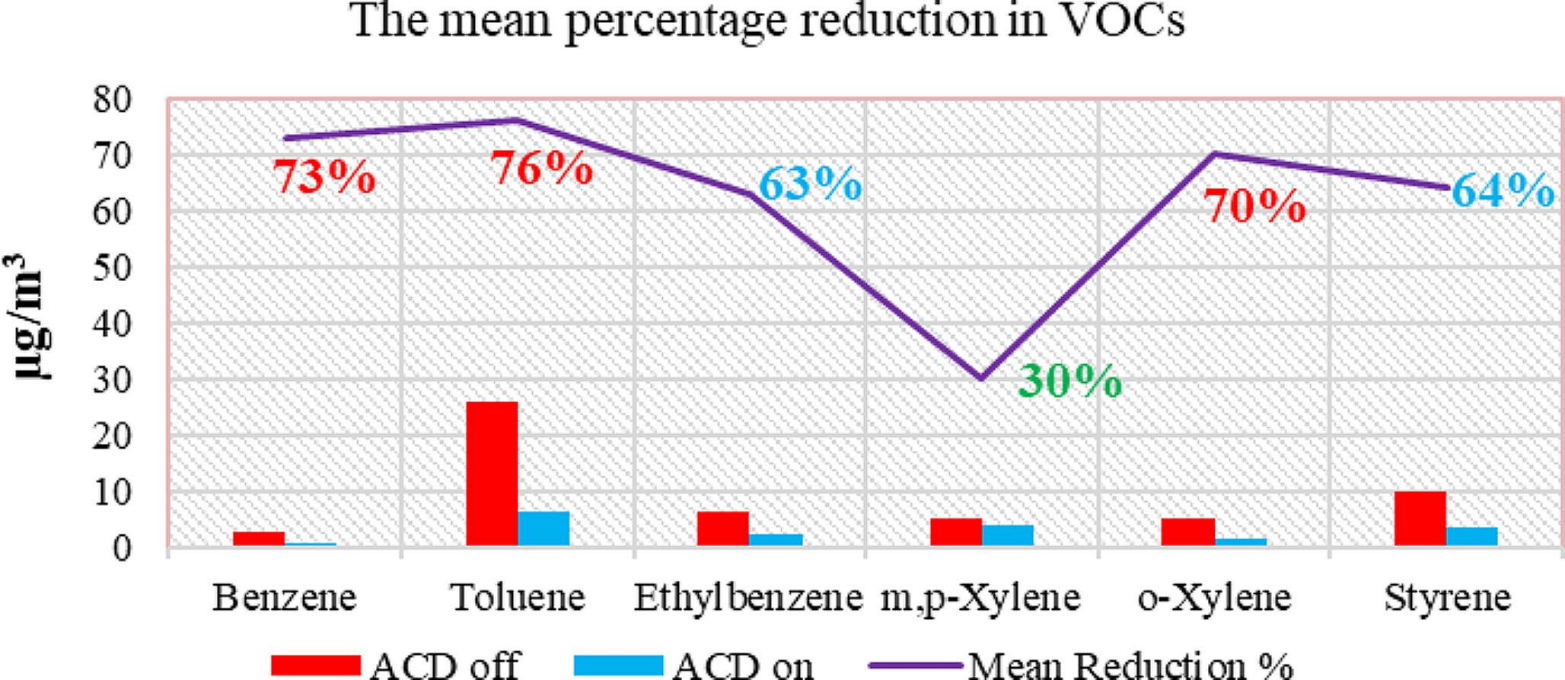
The mean percentage reduction in VOC concentrations when the ACDs were on/off

Statistical calculations were performed using the statistics package Statgraphics Centurion XV. One-way analysis of variance (ANOVA) was performed between the VOC concentration data with the air-cleaning devices when the air-cleaning devices were turned on, and the number of persons and patients in the ICU. There is a statistically significant difference in the 95.0% confidence between the concentrations of benzene (*p* = 0.01), toluene (*p* = 0.02), ethylbenzene (*p* = 0.02), styrene (*p* = 0.01), and m, p-xylene (*p* = 0.04) before the ACDs were installed and when the ACDs were turned on. The concentrations of VOCs were lower when the ACDs were on. Figure 3 shows the mean percentage reduction in VOC concentrations when the ACDs were on. There are not many articles available for comparing the results.

During the sampling, the number of persons in the ICU affected the concentration of VOCs. For example, in the morning, doctors, interns, and nurses were in the ICU to check up on the patients at about 10:00, washing/turning/transferring patients, and changing bedclothes, and cleaning the beds and floor was done. In the evening, the patients were asleep or resting and two or three nurses were in the ICU. The differences between concentrations of m, p-xylene, styrene, isopropylbenzene, n-propylbenzene, 1,2,4-trimethylbenzene, 4-isopropyl toluene, naphthalene, and total VOCs (TVOCs) and the number of patients were statistically significant. When the number of people in the ICU was more than 6 the concentration of VOCs was high.

### Compare the obtained data with other studies

In this study, OH radical-based ACD was utilized In literature, activated carbon filters are commonly used to reduce concentrations of VOCs. Carroll et al. (2022) used activated carbon filters in the operating room in their study. Observing a reduction of approximately 30% in VOCs when the device is on, and less than 1% when the device is off (Carroll and Kirschman 2022). Changing the activated carbon filter after 22 h of constant use showed an abrupt increase in the rate of toluene removal (Carroll and Kirschman 2022). In their study, Isinkaralar et al. (2023) used activated carbon filters to reduce VOCs. As a result, the amount of benzene, toluene, and xylene was reduced by 64% (Isinkaralar et al. 2023). The results of this study showed that the concentrations of benzene, toluene, and o-xylene decreased by approximately 70% after the air-cleaning devices were installed.

### Comparison of maximum criterion values to be provided in indoor environments for benzene and TVOCs in the EPA’s, WHO’s, ASHRAE’s, and Turkey’s safe-green buildings standard

**Fig. 4:**
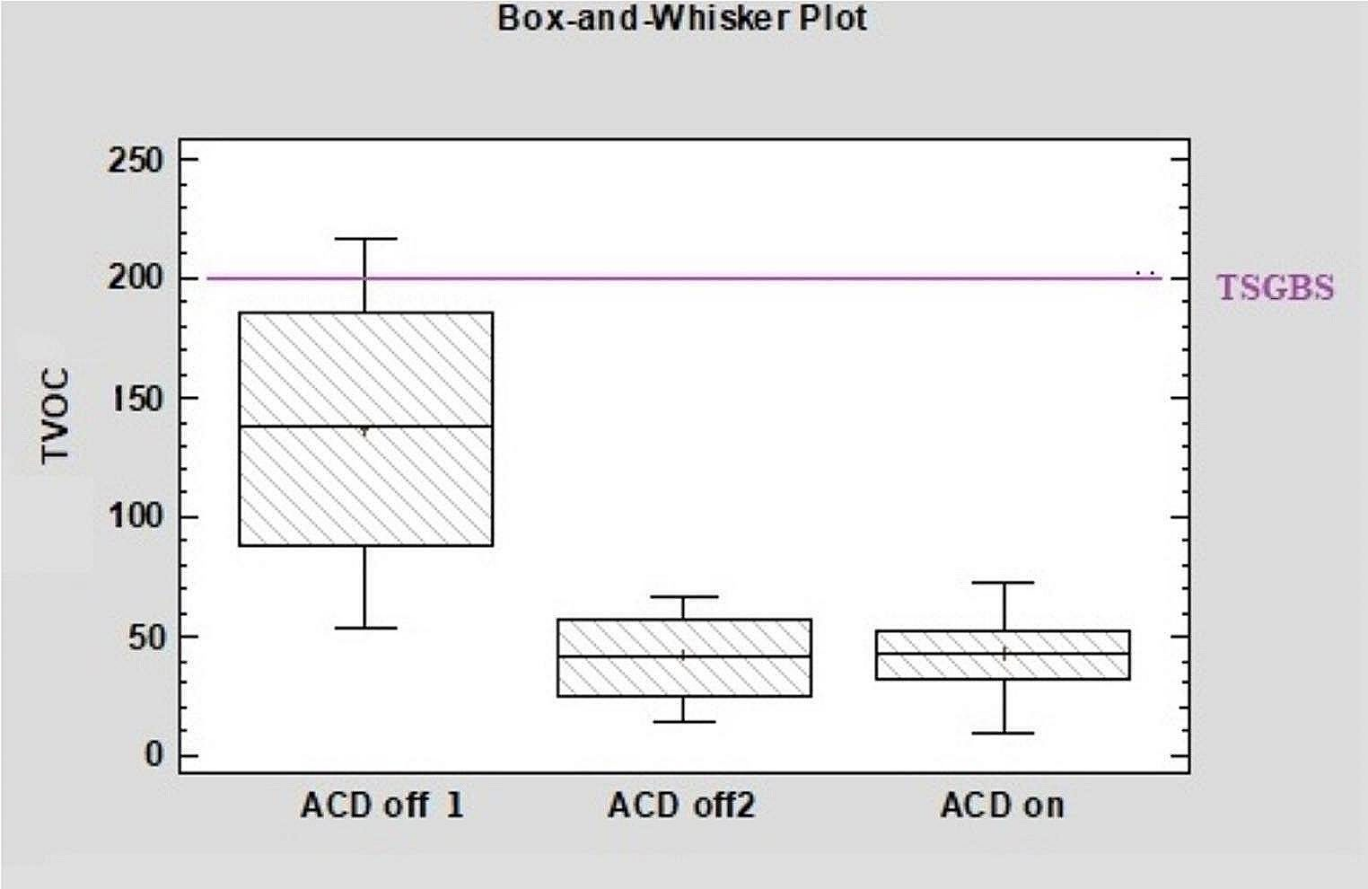
Box-and-whisker plots of the concentrations of TVOCs in the ICU (ACD 0ff 1, Before the devices were installed ACD off 2 When the devices were turned off after a two-week “on” period)

**Fig. 5:**
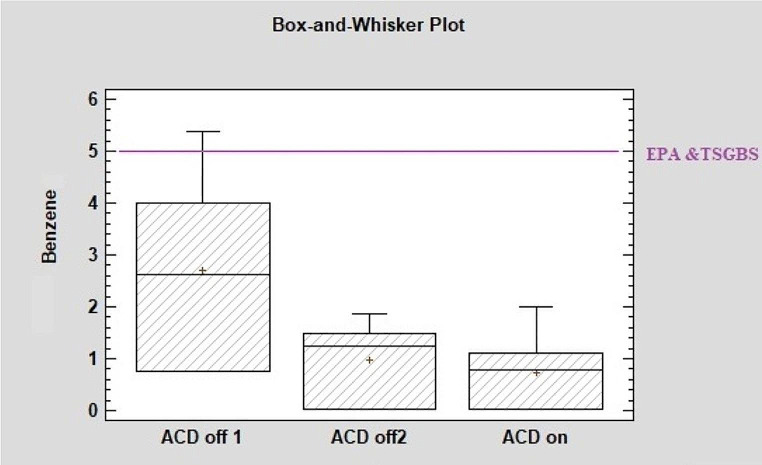
Box-and-whisker plots of the concentrations of benzene in the ICU (ACD 0ff 1, Before the devices were installed ACD off 2 When the devices were turned off after a two-week “on” period)

Box-and-whisker plots of the concentrations of indoor TVOCs (µg/m^3^) and benzene (µg/m^3^) were compared with the maximum criteria values for these materials in environments that should be provided for safe-green buildings determined by the Turkish Standards Institute (SGBTSI) (Lakestani 2015) and the EPA (Pickett and Bell 2011) (Figs. 4 and 5). The values accepted for TVOCs and benzene by the EPA, the American Heating-Cooling and Ventilation Engineering Society (ASHRAE), and the WHO are 500 µg/m3 and 5 µg/m^3^, respectively (Pickett and Bell 2011). For safe-green buildings in Turkey, the accepted indoor concentrations of TVOCs and benzene are 200 µg/m^3^ and 5 µg/m^3^.

The TVOC values obtained in the study were below the EPA, WHO, and ASHRAE values. Before the ACDs were installed, the maximum concentration of TVOCs (µg/m^3^) was greater than that in the Turkish safe-green buildings standard (TSGBS) (Fig. 4).

In the first week of sampling, the maximum concentration of benzene (µg/m^3^) was above the EPA and the TSGBS values (Fig. 5).

### Inhalation risk and health hazard risk assessment of VOCs in the ICU

The cancer risk that may arise due to inhalation of VOCs is calculated using information about the concentration of the pollutant in the air, how much people are exposed to this pollution level, what activities they engage in, and some personal characteristics such as age, sex, and weight. The risk of exposure to pollutants in the environment through air inhalation was determined using the equation below (Lim et al. 2014). For cancer and chronic hazard assessments, lifetime (70 years) is substituted for AT (EPA 2011). CR for healthcare personnel was calculated for 30 years and 8 h of work (Calabrese and Kenyon 2004; Dai et al. 2017).

1 $$Cancer{\text{ }}Risk{\text{ }}\left( {CR} \right){\text{ }} = {\text{ }}CDI{\text{ }} \times {\text{ }}IUR$$

CDI: Chronic daily intake (mg/kg/day).

IUR: Inhalation Unit Risk (µg/m^3^) ^**−1**^.

IUR amounts for VOCs are shown in Table 4 (IARC 2022). To calculate chronic daily pollution intake (CDI), the USEPA recommended using the following equation (EPA 2011):

2 $$CDI = C.ET.1{\text{ }}day/24{\text{ }}hours{\text{ }}EF{\text{ }}ED/AT$$

C: concentration of VOCs (µg/m^3^),

ET: exposure time (h/day) (8 h/day),

EF: exposure frequency (days/year) (260 days/year),

ED: exposure duration (years),

AT: average time (days),

**Table 4:** CR amounts and carcinogenic classification published by the international agency for research on cancer (IARC)

VOC	***IARC Group	Inhalation Unit risk (µg/m^3^)^−1^ (Reference)	Cancer Risk		
			^a^ACD off	ACD on	^b^ACD off
**Benzene**	1	6.0 × 10^− 6^ WHO*	5.69E-04	1.76E-04	2.18E-04
**Ethylbenzene**	2B	2.5 × 10^− 6^ OEHHA**	5.65E-04	1.61E-04	1.62E-04
**Naphthalene**	2B	8.7 × 10^− 5^ WHO*	1.57E-02	1.73E-03	1.36E-02

*WHO (World Health Organization), **OEHHA (Office of Environmental Health Hazard Assessment)*** The International Agency for Research on Cancer (IARC 2022) ^a^Before the devices were installed, ^b^When the devices were turned off after a two-week “on” period

In the present study, the cancer risk rate was divided into three classes: those with class A 10^− 4^ and above were included in the definite risk group, those with class B 10^− 4^-10^− 5^ were included in the probable risk group, those with class C 10^− 5^-10^− 6^ were included in the possible risk group, and class D less than 10^− 6^ were categorized as uncertain risk (Chen et al. 2016).

As can be seen, the average of naphthalene was in group A (a definite risk), while those of benzene and ethylbenzene were in the group B classification.

Non-carcinogenic is characterized risk calculated using the hazard index (HI). With this method, the concentration of non-cancer risk affecting human health in individuals exposed to BTEX is estimated. The HI is calculated by.

Equation (3):

3 $$HI = C/Rf$$

C: daily concentration (µg/m^3^),

RF: Reference dose for VOCs (µg/m^3^),

**Table 5:** Average Hazard Limit Value for VOCs

VOC	RF (µg/m^3^)	Hazard Index		
		^a^ACD off	ACD on	^b^ACD off
**Benzene**	9.6 ATSDR*	0.26	8.3E-2	1.03E-1
**Ethylbenzene**	1300 ATSDR*	4.73E-3	1.34E-3	1.35E-3
**Naphthalene**	3.7 ATSDR*	1.33	1.5E-1	1.16

*ATSDR (Agency for Toxic Substances and Disease Registry), **IRIS (Integrated Risk Information System) ^a^Before the devices were installed, ^b^When the devices were turned off after a two-week “on” period

If the average hazard limit value is greater than one, the concentration of that substance is above the maximum accepted value and poses a danger to human health. The acceptable level for HI from the US EPA (2004) is < 1 (Calabrese and Kenyon 2004). Considering the average values seen in Table 5, the hazard index value of naphthalene when the ACD was off was at a harmful level since it was greater than 1.

## Conclusion

This study is the first experiment on the monitoring of VOCs in an ICU in Turkey. The role of the concentration of VOCs in the ICU is important. It would be helpful to know which VOCs are most relevant to health. In this study, the concentrations of VOCs were reduced when ACDs were turned on. The number of healthcare personnel and patients during the sampling affected the concentrations of VOCs. As a result of the study, it was observed that the concentrations of VOCs were high in the morning because that was when doctors, interns, and nurses were in the ICU to examine the patients. The percentages of BTEX and styrene were reduced after ACDs were installed. Cancer risks were calculated for 3 VOCs (Table 3) and the average of naphthalene was observed in the definite risk group (A). The hazard index of naphthalene at the first and fourth weeks when ACDs were off was measured at the harmful levels. When the healthcare professionals working in the ICU were asked how they felt when the ACDs were on and off, they said they suffered from less coughing and fewer headaches when the ACDs were turned on.

In hospitals, especially in the ICUs and operating rooms, the use of chemical products is the primary source of contamination. A high number of products, such as cleaning and disinfectant products, alcohol-based products, anesthetic gases, and laboratory products, are used for disinfection, sterilization, and other activities (Bessonneau et al. 2013). In addition, the healthcare personnel were informed about the sources of pollutants associated with health problems and measures to reduce them. The reduction of hospital air pollution through ACDs in a hospital environment has an extensive impact on society by reducing expenditures on health.

## Data Availability

Data available on request from an author.
